# The double S technique to achieve aesthetic flat closure after conventional mastectomy

**DOI:** 10.1186/s12957-022-02515-3

**Published:** 2022-02-21

**Authors:** Daniel Steffens, Elisabeth A. Kappos, Alexander Lunger, Fabienne D. Schwab, Lea Zehnpfennig, Walter Paul Weber, Martin Haug, Viola Heinzelmann-Schwarz, Christian Kurzeder

**Affiliations:** 1 Department of Gynecology and Gyn Oncology, Hospital for Women, University Hospital Basel, Basel, Switzerland; 2 Breast Centre, University of Basel, Basel, Switzerland; 3 Department of Plastic, Reconstructive, Aesthetic and Hand Surgery, University of Basel, Basel, Switzerland; 4 Department of Breast Surgery, University of Basel, Basel, Switzerland

**Keywords:** Mastectomy, Dog ear, Axillary skin flaps, Flat closure

## Abstract

**Background:**

Lateral excess tissue after mastectomy is a frequent problem, which should be included into preoperative planning. Women with lateral tissue abundance are frequently impaired cosmetically and functionally. We suggest a novel oncoplastic mastectomy technique to eliminate the above mentioned.

**Methods:**

Surgical technique

Two small horizontal lines are drawn, one line above and one line below the Nipple Areola Complex. These lines should represent the possible skin excision and allow tight skin closure. Consecutively, two ending points of the incision are planned, one close to the xyphoid area and the other one in the anterior axillary line. These points are then interconnected in an s-shaped manner to form a double s-shaped skin excision.

**Results:**

The double S-shaped technique is an easy reproducible technique which not only allows good access to the lateral side of the mastectomy, but also and mainly the reduction of lateral fat and skin.

**Conclusion:**

The double S mastectomy allows for simultaneous removal of access in the axillary region, eliminating skin, and fat as needed and preventing the lateral dog ear

## Introduction

**Fig. 1:**
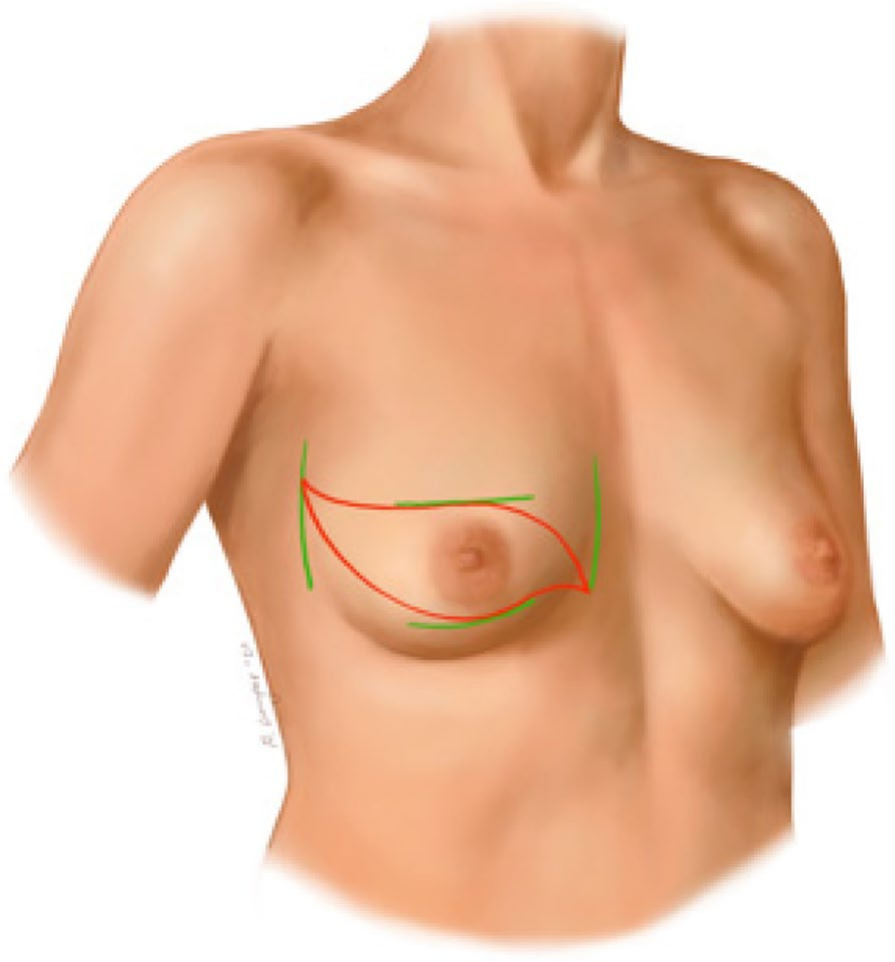
Incision lines

**Fig. 2:**
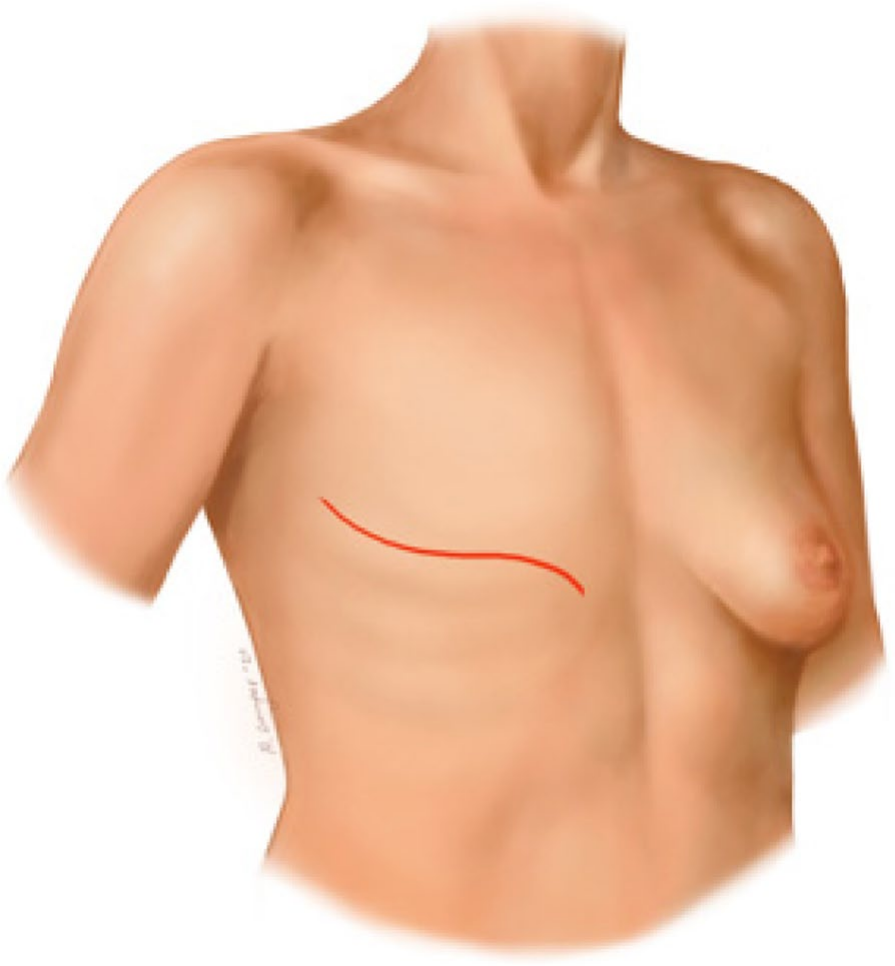
Final scar

**Fig. 3:**
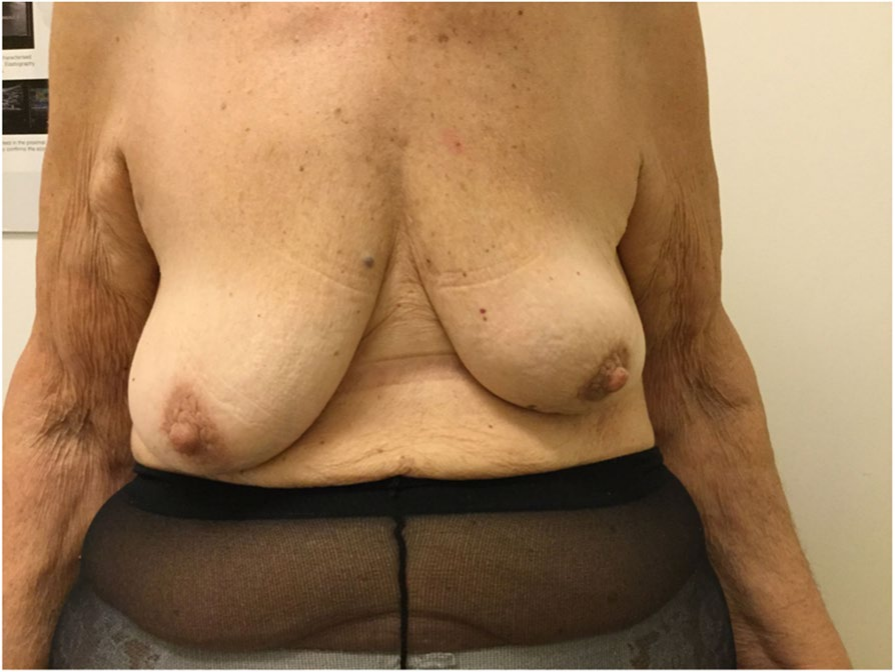
Pre-operative

**Fig. 4:**
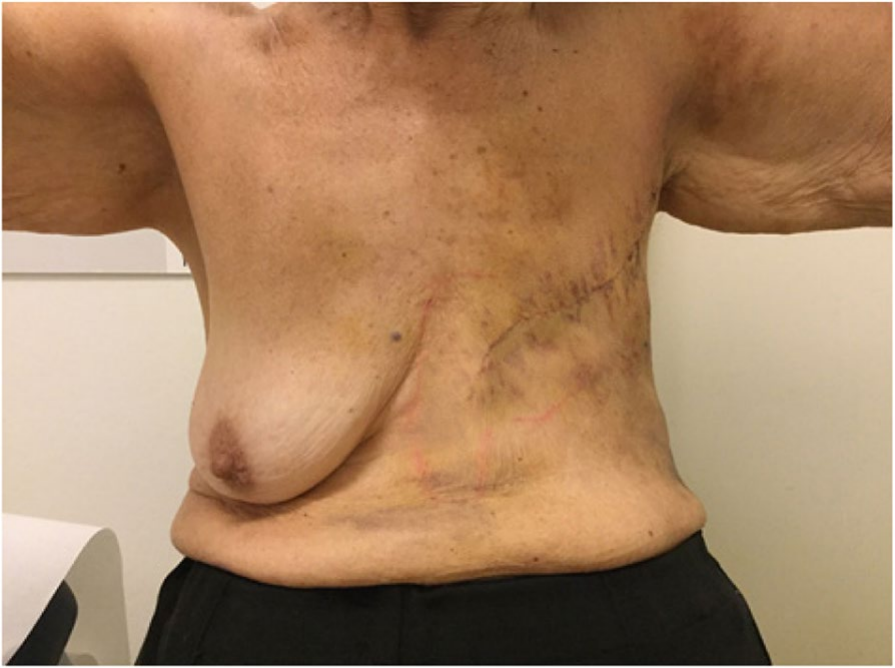
Post-operative

**Fig. 5:**
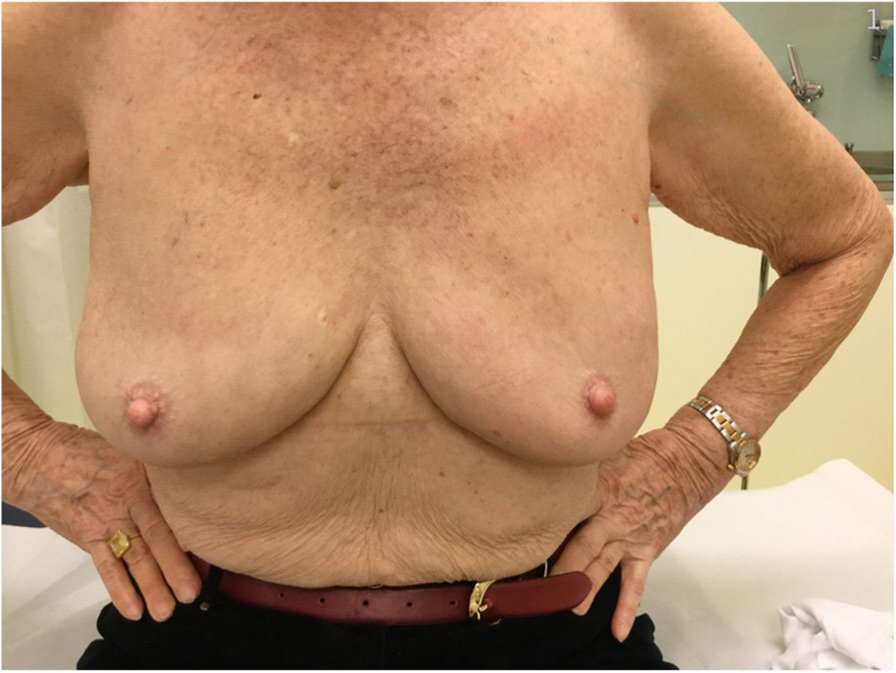
Pre-operative

**Fig. 6:**
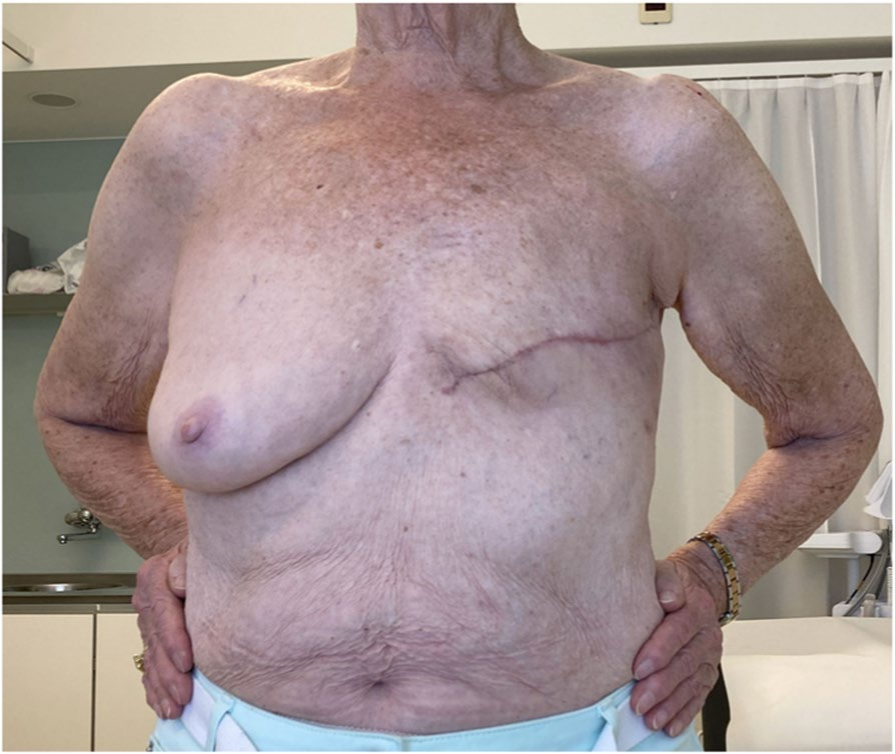
Post-operative

**Fig. 7:**
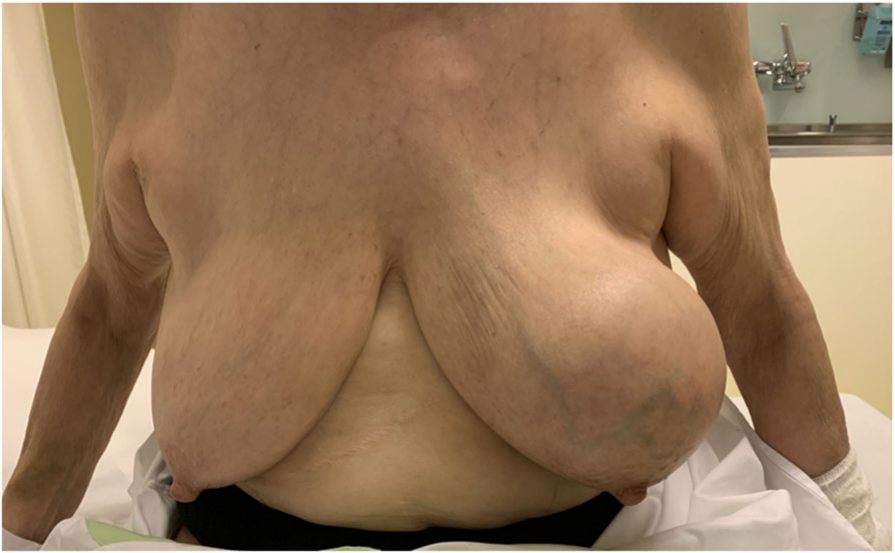
Pre-operative

**Fig. 8:**
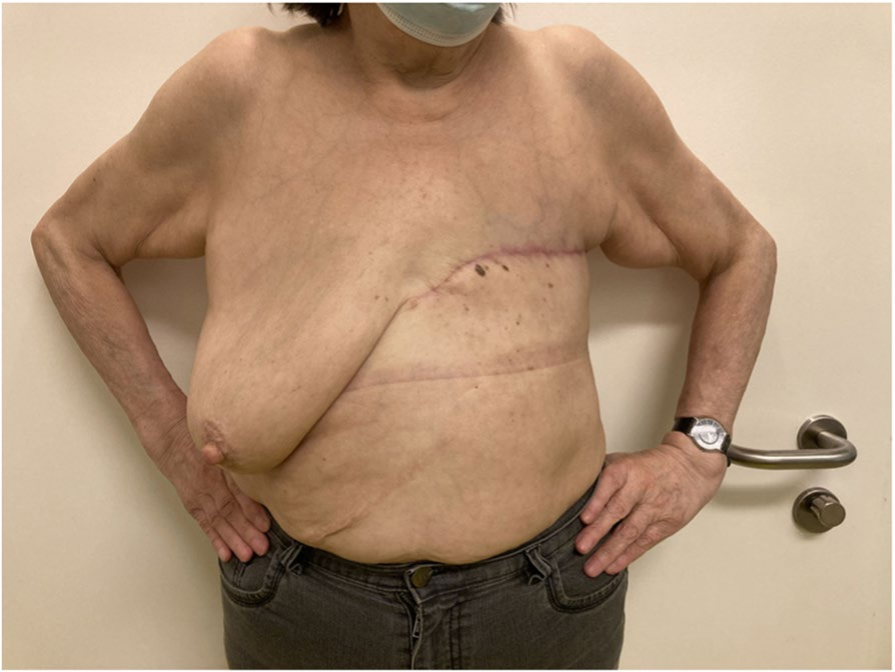
Post-operative

Breast cancer (BC) is the most common cancer among women worldwide [1]. Due to major improvements in BC treatment within the last decades, mortality of early-stage BC patients has significantly decreased. Therefore, patient’s health-related quality of life (QoL) and esthetical satisfaction has become a major focus of breast cancer therapy [2] Figs. 1, 2, 3, 4, 5, 6, 7, 8.

Mastectomy is an important operation technique in BC. However, it is well established that total mastectomy might have a negative impact on women QoL and psychosexual well-being [3–6].

Nevertheless, some women elect to forgo reconstruction despite adequate information and access to appropriate options for reconstruction [7]. Several motivations for not pursuing reconstruction were identified including concerns regarding placement of a foreign body, necessity for additional procedures, as well as the belief that reconstruction was not important. In fact, the going flat movement (mastectomy without reconstruction) is growing and the going flat population seeks empowerment regarding their choice to go flat by raising awareness of flat denial among surgeons and demanding competence to achieve aesthetic flat closure [8]. Recent data suggest high patient satisfaction after going flat [9] and the Oncoplastic Breast Consortium recently included the pursuit of aesthetic flat closure into its mission statement [10].

For those groups of patients receiving a conventional mastectomy without breast reconstruction, precise planning of the mastectomy incision is of particular importance to achieve aesthetic flat closure and, accordingly, satisfaction with the surgical outcome. The majority of breast cancer patients undergoing mastectomy will have transversely oriented incisions over the chest wall, created by elliptical incisions made superior and inferior to the nipple-areolar complex. Redundant skin and fat tissue can result in so-called dog ears in the axilla and next to the sternum and can lead to inferior cosmetic results and cause considerable discomfort and dissatisfaction.

Here, we want to describe a new versatile oncoplastic technique, the double S oncoplastic mastectomy, to avoid excessive axillary tissue and dog ears. It allows for resection of variable amounts of axillary skin and subcutaneous fat tissue and can be particularly useful in patients with lateral fat and skin abundance.

## Methods/description of the surgical technique

Two small horizontal lines are drawn, one line above and one line under the nipple areola complex (NAC). These lines should mark the width of the skin segment to be resected and allow for tight skin closure. Then, two terminal landmarks of the incision are planned, one close to the xyphoid area and the other one in the anterior axillary line region. The final skin incision lines are then completed to delineate a double s-shaped segment. Both terminal points can be shifted medially and laterally in order to eliminate excessive skin and fat tissue if needed.

## Results

The double S-shaped technique is an easy reproducible technique which not only allows good access to the lateral side of the mastectomy, but also and mainly the reduction of lateral fat and skin.

## Discussion

Despite great achievements in oncoplastic breast-conserving surgery, mastectomy with and without reconstruction remains an integral part of the surgical armamentarium for breast cancer therapy. A significant fraction of patients will elect to forgo reconstructive surgery and decide to go flat. A recent survey of 931 patients within the growing online going flat communities found that only 74.1% of patients were satisfied with their surgical results [11]. Dissatisfaction was more likely reported among women with a body mass index (BMI) above 30 kg/m^2^, indicating that aesthetic issues with flaps of excessive skin or subcutaneous tissue might play a major causal role, but this is not scientifically proven yet. Dog ears have been described as a result of inadequate tissue resection at the lateral and medial ends of the incision. A recent survey on social media could demonstrate that avoiding dog ears is a crucial contributor to patients’ satisfaction [1]. Multiple modified techniques of the transversely oriented incision have already been proposed.

Farrar et al published 1988 the Y closure to address the issue with the lateral dog ear [6]. Several publications concerning the Y closure or so-called fish tail plasty closure have been published since [3, 4, 12, 13]. These techniques allow for resection of the accessory fat tissue by incorporation of two additional scars and an angle which increases the risk of wound healing complications.

To avoid the risk of skin necrosis the “tear drop incision” was described with the aim to avoid additional scars. This incision technique produces a linear footprint without additional scars and allows for good access to the axillary fat pad. However, due to variable incision lines, depending on the tissue to be resected, the disadvantages could be variable results depending on the point of wound closure of the lateral apex of the incision [14]. Thomas et al. [15] described the waisted teardrop technique, to improve the technique by Mirza et al. [14]. He retracted the medial end of the tear drop ellipse laterally and the lateral end of the ellipse medially. After this, the symmetry of the ellipse is then reconstituted and marked. For obese patients, he adds an advancement of the lateral flap, which is de-epithelialized and subsequently sutured into the mastectomy wound. The cosmetic outcome showed acceptable results with no additional scars, but with this technique the surface of the wound is significantly enlarged and also wound puckering might occur [15]. Similar concerns have been discussed with a modified technique by Devalia et al. as described in 2007 [16].

A different approach for the lateral dog ear is the extending of the ellipse incision into the lateral redundant skin which was described by Clough 2012 [17] with a “L”-shaped incision. This technique allows good access to the axilla without additional scars and good elimination of the lateral dog ears. If there is too much tension on the edges of the wound, especially axillary, or removal of too much skin this could lead to wound healing disturbances [5].

2018 E.L. Hill et al. [18] described an “angel wing” technique where the incision is drawn medially to laterally using the standard surgical width to length ratio of 1–3 and extending to the lateral redundant skin and fat as angel wing. The pros are that it is easy to perform, has no additional scars, and has good motion of the arm. The cons could be the extent of the scar and longer duration of the operation [18].

Here, we describe a new versatile technique, the double S mastectomy technique which provides superior access to axillary tissue and allows the resection of variable amounts of excessive tissue at the medial and lateral ends. The double S mastectomy also offers superior exposure without necessity for extension of the incision when broad access to the axillary compartment is sought. The lateral dog ear will effectively be prevented without further scars. In addition, the double S incision can be adapted to the tumor localization when eccentric skin resection is required. It is an easy technique to reproduce with good cosmetic results if planned properly.

Up to date, only few surgical techniques have been evaluated with respect to impact on quality of life and patient satisfaction. Prospective validation of the proposed technique for aesthetic flat closure should therefore be pursued using objective and subjective aesthetic scales. No firm conclusions can be drawn considering the retrospective nature and limited amount of data describing the double S technique.

According to the literature currently there is no standard incision and a plethora of surgical techniques to prevent the lateral dog ear had been published. The double S technique adds one more option with cosmetic emphasis on a flat chest in case aesthetic flat closure is the patient’s first choice.

## Conclusion

Lateral excess of skin and fat can cause major discomfort and dissatisfaction for the mastectomy patient. We suggest an easy reproducible technique to avoid the later and achieve high patient satisfaction.

## Data Availability

Not applicable

## Abbreviations

BC: Breast cancer
QoL: Quality of life
NAC: Nipple areola complex
BMI: Body mass index
